# Dr. Hunter’s Improvement

**Published:** 1852-04

**Authors:** 


Dr. Hunter's Improvement.—We have recently been shown several specimens of Dr. Hunter's improved method of setting artificial teeth. This imment consists in a cement and gum which unites into one block as many teeth as he may wish, even to a full upper or lower denture. Single teeth are selected and arranged to a narrow platina base. The width of the base of the teeth and gum, and which is swedged to the metal cast, and covers the alveolar border just where the artificial denture is to rest. A thin platina strip of plate is bent to suit the circle of the teeth, and attached to the lower pins of the teeth, and wide enough to come in contact with the platina base. The teeth are thus arranged and articulated to suit, and the cement is then adjusted around the labial border and between the teeth just as desired. In this condition it is baked, and then enamelled with gum enamel. The body unites the teeth into one block, and is as much superior to the old method of making block teeth as the American teeth are superior to the French. The gum which the Dr. uses he says will not contract by heating, and the specimens we have seen show no evidence of this having taken place in cooling. The practical dentist will at once see the importance of this, for the teeth and gum being adapted to the plate which fits the mouth, if no contraction takes place, it will also fit after it comes out of the furnace, and is ready for gold backings, which cover the platinum straps, and are united to the upper pin of the teeth, and when soldered unites with this and the platinum straps and also the gold plate, making, when done, a very neat and substantial operation. By this process a much more perfect articulation is obtained than in ordinary block work—and the set instead of being in sections is in one block.

The specimens which the Dr. sent to the World's Fair were put up we believe much as we have described. One of these was put up on a platina plate, and one on silver, which was covered with gold by the galvanic process. One of these sets, the Dr. made first the teeth to represent the natural organs, fang and crown, then cut off the fang to suit the length of the tooth on the plate, and as before described, united the whole with body and enamel.

We have given to some extent a description of Drs. Allen's and Hunter's method of fastening teeth to plates, and to each other. Either method we regard as a great improvement, and worthy the attention of the profession. We are glad that our city can lay claim to both these improvements. They are however, excepting in detail, almost the same thing, both unite teeth in one continuous block, and both have, as we suppose, overcome shrinkage. Both have we believe been at work for years to effect this. As it regards priority of discovery, we pretend not to decide, yet we do not believe that either received any aid or information of the other. Both have, as we believed, arrived at the present perfection of their plans without the assistance of any one, excepting such information as they might glean from authorities, such as Ure, Delabarre, and those in our country who are engaged in the manufacture of teeth.

Dr. Allen, it is known, has obtained a patent right on his improvement, and we believe is making every effort to introduce it into practice, and charges for instruction and right from $150 up to a larger amount, depending on circumstances. Dr. Hunter offers to instruct for about the same, provided a class is made up for that purpose, so that in either case the profession must expect to pay for the knowledge. Of this we do not complain, for we would prefer paying either the amount asked to working it out ourselves, and we say this even if they would give us their recipes. There is much in the manipulation almost as essential as the other, at least both are essential to success. We should have been glad however, if both could have given the profession the benefit of their improvement without any special tax.

